# The effect of breast MRI on disease-free and overall survival in breast cancer patients: a retrospective population-based study

**DOI:** 10.1007/s10549-020-05906-w

**Published:** 2020-09-15

**Authors:** T. J. A. van Nijnatten, L. P. T. van Tiel, A. C. Voogd, C. G. M. Groothuis-Oudshoorn, S. Siesling, M. B. I. Lobbes

**Affiliations:** 1 Department of Radiology and Nuclear Medicine, Maastricht University Medical Center+, P.O. Box 5800, 6202 AZ Maastricht, The Netherlands; 2 GROW – School for Oncology and Developmental Biology, Maastricht University Medical Center+, Maastricht, The Netherlands; 3 Department of Health Technology and Services Research, Technical Medical Center, University of Twente, Enschede, The Netherlands; 4 Department of Research and Development, Netherlands Comprehensive Cancer Organisation, Utrecht, The Netherlands; 5 Department of Epidemiology, Maastricht University Medical Center+, Maastricht, The Netherlands; 6 Department of Medical Imaging, Zuyderland Medical Center, Sittard-Geleen, The Netherlands

**Keywords:** Breast cancer, Magnetic resonance imaging, Disease-free survival, Overall survival

## Abstract

**Purpose:**

To evaluate the effect of breast MRI on overall survival (OS) and disease-free survival (DFS) of patients with invasive breast cancer in the Netherlands.

**Methods:**

We selected all women from the Netherlands Cancer Registry diagnosed with invasive breast cancer (a) between 2011 and 2013 for the OS-cohort and (b) in the first quarter of 2012 for the DFS-cohort. The study population was subdivided into an MRI and non-MRI group. In addition, subgroups were created according to breast cancer subtype: invasive carcinoma of no special type (NST) versus invasive lobular carcinoma (ILC). OS and DFS were compared between the MRI and non-MRI group using the Kaplan–Meier method and the log-rank test. Cox proportional hazard regression analysis was performed to estimate hazard ratios (HR) with a 95% confidence interval (CI). To account for missing data, multiple imputation was performed.

**Results:**

Of the 31,756 patients included in the OS-cohort (70% non-MRI and 30% MRI), 27,752 (87%) were diagnosed with invasive carcinoma NST and 4004 (13%) with ILC. Of the 2464 patients included in the DFS-cohort (72% non-MRI and 28% MRI), 2161 (88%) were diagnosed with invasive carcinoma NST and 303 (12%) with ILC. The distribution of breast MRI use was significantly lower over different age categories, from 49.0% aged < 50 to 16.5% aged > 70. Multivariable Cox regression showed that breast MRI was not significantly associated with OS overall (HR 0.91, 95%-CI 0.74–1.11, *p* = 0.35), nor in the different histological subtypes. Multivariable Cox regression analysis showed that breast MRI was also not significantly associated with DFS (HR 1.16, 95%-CI 0.81–1.67), nor in the different histological subtypes.

**Conclusion:**

Use of breast MRI was not significantly associated with an improved OS or DFS in patients treated with primary surgery.

**Electronic supplementary material:**

The online version of this article (10.1007/s10549-020-05906-w) contains supplementary material, which is available to authorized users.

## Introduction

Breast cancer is the second most common cancer-type in the world (2.09 million cases) and for women it is the most common cancer-type [1]. Annually, more than 15,000 Dutch women are diagnosed with breast cancer, the most frequent type of cancer among women in the Netherlands [2].

Conventional imaging techniques for early detection and diagnosis of breast cancer are full-field digital mammography (FFDM) and ultrasound, in combination with tissue sampling of suspicious lesions [3]. In the last decades, an increase is observed in the use of (preoperative) breast magnetic resonance imaging (MRI) as an additional diagnostic imaging technique, in particularly applied in cases where the therapeutic pathway is still questioned based upon conventional imaging. However, the use of breast MRI has become a subject of debate as several studies questioned its additional value, even arguing that it might result in more mastectomies [4]. Theoretically, breast MRI could have beneficial effects, because of its superior ability to assess for instance maximum tumour diameter, multifocality, and presence of contralateral breast cancer [5, 6]. Therefore, breast MRI could be used for optimizing the extent of surgery and radiotherapy, which may reduce the risk of involved surgical margins and the need for re-excision, moreover it may result in improved local control [7], which in turn might lead to fewer metastases, and an improved disease-free (DFS) and overall survival (OS). On the other hand, due to the high sensitivity of MRI, the possibility of overdiagnosis exists and it might even result in a higher risk of false-positive results [3]. Both may lead to unnecessary and/or more extensive resections, treatment delay, and higher costs [8, 9].

Regarding short-term effects of the use of breast MRI, previous studies have shown that mastectomy rates increased, and that the risk of positive surgical margins and re-excision rates showed no or only a slight improvement [3, 4, 8]. However, for some subgroups, such as invasive lobular carcinoma (ILC), breast MRI might have beneficial short-term effects, but published results are contradictory [3, 4, 8]. Regarding the long-term effects of breast MRI, previous studies have shown that there were no statistically significant differences in the risk of local and distant recurrences, contralateral breast cancer development, and disease-free and overall survival [7, 9–14]. However, some studies indicated that breast MRI has a tendency towards improved survival [11, 14]. Hence, there are still uncertainties about whether breast MRI has a beneficial effect on the long-term outcome. The purpose of this retrospective population-based study was to evaluate the effect of the use of breast MRI on the overall and disease-free survival of patients with invasive breast cancer in the Netherlands.

## Methods

### Data collection

This retrospective study included all female patients treated with surgery for invasive breast carcinoma of no special type (NST) or ILC, diagnosed in the period of 2011–2013 in the Netherlands. The OS had a maximum follow-up of 7 years. Data on recurrent disease for the DFS-cohort were available for patients diagnosed and treated in the first three months of 2012, and had a maximum follow-up of 5 years. No age-limits were applied and patients treated with adjuvant radiotherapy, chemotherapy, hormonal and/or target therapies were included. Patients who did not undergo surgical treatment, who had distant metastases at baseline, and/or who had received neo-adjuvant therapy were excluded.

Data were obtained from the Netherlands Cancer Registry (NCR), and were registered by trained registrars based on notification by the automated pathology laboratory archive (PALGA). The 7th edition of the TNM-classification was used [15]. According to national guidelines, routine use of breast MRI was not recommended, but could be considered in these indications: (a) patients with ILC, (b) patients with invasive carcinoma who preferred breast conserving surgery but who had discrepancies in tumour size assessment between physical examination and imaging. These recommendations applied in particular to young women [16].

The following variables were selected for the present study: age at diagnosis (categorized in < 50, 50–59, 60–69, or ≥ 70 years), tumour status (T1, T2, or T3-4), nodal status (N0, N1, N2, or N3), molecular subtypes (ER/PR + HER2−: estrogen receptor (ER) and/or progesterone receptor (PR) positive, HER2 negative; ER/PR + HER2 + : ER and/or PR positive, HER2 positive; HER2 enriched: ER and PR negative, HER2 positive; and triple negative: ER, PR, and HER2 negative), histological grade (low, medium, or high), multifocality (yes or no), tumour location (lateral, medial, or other), breast MRI use (yes or no), surgical margin status after surgery (negative margin (NM) < 0 mm, focal positive margin (FPM) < 4 mm, or more than focal positive margin (MFPM) > 4 mm), type of final surgery (mastectomy or breast conserving surgery), adjuvant therapy (radiotherapy, chemotherapy, hormonal or targeted treatment), and vital status (date of death). For patients diagnosed and treated in the first quarter of 2012, active follow-up was conducted whereby information on the date of local recurrence (yes or no), regional recurrence (yes or no), and distant metastases (yes or no) was collected. Time period until death or last contact was linked with the database of the municipality, and was updated until February 1st, 2018.

### Statistical analysis

The study population was divided into an MRI and non-MRI group, according to the use of breast MRI. Subsequently, the study population was stratified into one of the following histological subgroups: invasive carcinoma NST or ILC. General characteristics between the groups were tested using the Chi-square test for categorical variables and the Mann–Whitney *U* test for continuous variables. OS and DFS were calculated with the Kaplan–Meier method and its survivor function, which predicts the chance of survival after a given period of time. Kaplan–Meier survival curves were compared with the log-rank test. For the DFS an event was defined as any local recurrence (LR), regional recurrence (RR), or distant metastasis (DM) within 5 years after the primary diagnosis. Possible confounders were examined using univariable and multivariable Cox proportional hazard regression analysis, with hazard ratio (HR) and corresponding 95% confidence interval (CI). In the multivariable model of the OS analysis, the interaction term MRI and age per category was included to test whether the effect of MRI differs between age category. Multiple imputation by the chained equations, with 50 iterations and 20 imputations, was used to account for missing data [17]. The pooled results were used based on Rubin’s rule. Variables in the multivariable model, that were not statistically significant, were excluded based on significance and the Akaike’s information criterion (AIC) selection method. Proportional hazards assumption was tested by the Schoenfeld test and by plotting the scaled Schoenfeld residuals. Statistical analyses were performed by using Stata/SE version 14.2 (StataCorp. LP, College Station, TX, USA). *P*-Values (two-sided) less than 0.05 were considered statistically significant.

## Results

### General characteristics

In the period 2011–2013 31,877 patients were treated with surgery for invasive carcinoma NST or ILC. Patients with no follow-up data (*n* = 80), unknown surgery (*n* = 20), or an unknown adjuvant treatment (*n* = 21) were excluded. After exclusion of these patients 31,756 (99.6%) patients composed the OS-cohort for the analysis of the OS. Of these patients 22,124 (70%) did not undergo breast MRI, 27,752 (87%) had invasive carcinoma NST (73% non-MRI and 27% MRI) and 4004 (13%) had ILC (44% non-MRI and 56% MRI). The final study cohort for the DFS (DFS-cohort) included 2464 patients, of whom 1767 (72%) did not undergo breast MRI. Of these patients, 2161 (88%) had invasive carcinoma NST (75% non-MRI and 25% MRI) and 303 (12%) had ILC (46% non-MRI and 54% MRI).

Table 1 shows patient, tumour, and treatment characteristics of the OS-cohort, according to breast MRI use. Patients in the non-MRI group were older compared to the MRI group (62.9 vs 56.1 years, respectively) and were less often treated with adjuvant chemotherapy, hormonal and/or target therapies. In addition, the percentage of patients who underwent breast MRI was lower in higher age categories (from 49.0% in age < 50 until 16.5% in age > 70). The MRI group had slightly larger tumours, were more likely to have axillary lymph node involvement and multifocal tumours, and more often underwent mastectomy as the final surgical procedure. The histological subgroups had similar patient characteristics, except for the ILC subgroup, where, in general, more mastectomies as final surgery were performed. Regarding the involvement of surgical margins after breast conserving surgery, there was no statistically significant difference between the MRI and non-MRI group for the total study population (*p* = 0.08) and the invasive carcinoma NST (*p* = 0.39). For the ILC subgroup a statistically significant difference was observed (*p* = 0.002); here the MRI group was more likely to have negative margins (79% versus 73%), and less likely to have more than focal positive margins (8% versus 12%), compared to the non-MRI group.

**Table 1:** Patient, tumour, and treatment characteristics total study population and per subgroup, according to use of breast MRI

Characteristic	Total study population				Invasive carcinoma NST				ILC			
	non-MRI (*n* = 22,124)		MRI (*n* = 9632)		non-MRI (*n* = 20,366)		MRI (*n* = 7386)		non-MRI (*n* = 1758)		MRI (*n* = 2246)	
	*n*	(%)	*n*	(%)	*n*	(%)	*n*	(%)	*n*	(%)	*n*	(%)
*Age*												
Mean (range)	62.9	(19–97)	56.1	(21–94)	62.5	(19–97)	54.8	(21–94)	66.9	(28–93)	60.3	(24–88)
< 50	3092	(14)	2976	(30.9)	2936	(14.4)	2557	(34.6)	156	(8.9)	419	(18.7)
50–59	5375	(24.3)	2821	(29.3)	5083	(25)	2168	(29.4)	292	(16.6)	653	(29.1)
60–69	7078	(32)	2534	(26.3)	6529	(32.1)	1834	(24.8)	549	(31.2)	700	(31.2)
> 70	6579	(29.7)	1301	(13.5)	5818	(28.6)	827	(11.2)	761	(43.3)	474	(21.1)
*Tumour status*												
1	15,366	(69.8)	6223	(65)	14,444	(71.2)	5072	(69.1)	922	(52.7)	1151	(51.5)
2	6115	(27.8)	2919	(30.5)	5446	(26.9)	2074	(28.2)	669	(38.2)	845	(37.8)
3–4	547	(2.5)	436	(4.6)	388	(1.9)	199	(2.7)	159	(9.1)	237	(10.6)
Unknown	96		54		88		41		8		13	
*Nodal status*												
0	14,831	(68.5)	6168	(64.7)	13,748	(69)	4736	(64.8)	1083	(63.5)	1432	(64.5)
1	5313	(24.6)	2547	(26.7)	4886	(24.5)	1989	(27.2)	427	(25)	558	(25.1)
2	928	(4.3)	527	(5.5)	842	(4.2)	396	(5.4)	86	(5)	131	(5.9)
3	566	(2.6)	288	(3)	456	(2.3)	188	(2.6)	110	(6.4)	100	(4.5)
Unknown	486		102		434		77		52		25	
*Histological grade*												
Low	5417	(25.3)	2112	(22.8)	5167	(26.2)	1726	(24.2)	250	(14.9)	386	(18)
Medium	9690	(45.2)	4614	(49.8)	8423	(42.7)	3023	(42.4)	1267	(75.3)	1591	(74.1)
High	6316	(29.5)	2547	(27.5)	6150	(31.2)	2378	(33.4)	166	(9.9)	169	(7.9)
Unknown	701		359		626		259		75		100	
*Multifocal*												
No	19,579	(89.5)	7305	(76.1)	18,118	(89.9)	5630	(76.5)	1461	(84.8)	1675	(74.9)
Yes	2300	(10.5)	2289	(23.9)	2039	(10.1)	1728	(23.5)	261	(15.2)	561	(25.1)
Unknown	245		38		209		28		36		10	
*Molecular subtype*												
ER/PR + HER2-	16,531	(77.3)	7376	(78.3)	14,976	(76)	5300	(73.3)	1555	(93)	2076	(94.4)
ER/PR + HER2 +	1707	(8)	836	(8.9)	1651	(8.4)	757	(10.5)	56	(3.3)	79	(3.6)
HER2 enriched	808	(3.8)	372	(3.9)	801	(4.1)	359	(5)	7	(0.4)	13	(0.6)
Triple negative	2327	(10.9)	842	(8.9)	2273	(11.5)	811	(11.2)	54	(3.2)	31	(1.4)
Unknown	751		206		665		159		86		47	
*Tumour location*												
Lateral	10,708	(49.0)	4302	(45.2)	9863	(49.0)	3340	(45.7)	845	(48.9)	962	(43.4)
Medial	4693	(21.5)	1830	(19.2)	4410	(21.9)	1455	(19.9)	283	(16.4)	375	(16.9)
Other	6462	(29.6)	3389	(35.6)	5861	(29.1)	2510	(34.4)	601	(34.8)	879	(39.7)
Unknown	261		111		232		81		29		30	
*Final operation*												
Mastectomy	7987	(36.1)	4717	(49)	6981	(34.3)	3441	(46.6)	1006	(57.2)	1276	(56.8)
BCS	14,137	(63.9)	4915	(51)	13,385	(65.7)	3945	(53.4)	752	(42.8)	970	(43.2)
*Surgical margin*¥												
NM	20,207	(93.2)	8955	(93.7)	18,650	(93.4)	6899	(94.1)	1557	(91.7)	2056	(92.4)
FPM	1286	(5.9)	530	(5.5)	1174	(5.9)	391	(5.3)	112	(6.6)	139	(6.3)
MFPM	178	(0.8)	73	(0.8)	149	(0.7)	44	(0.6)	29	(1.7)	29	(1.3)
Unknown	453		74		393		52		60		22	
*Adjuvant therapy*												
Radio—yes	15,456	(69.9)	6129	(63.6)	14,452	(71)	4728	(64)	1004	(57.1)	1401	(62.4)
Chemo—yes	7466	(33.7)	4760	(49.4)	7031	(34.5)	3,796	(51.4)	435	(24.7)	964	(42.9)
Hormonal—yes	11,946	(54)	5995	(62.2)	10,678	(52.4)	4267	(57.8)	1268	(72.1)	1728	(76.9)
Target—yes	1643	(7.4)	969	(10.1)	1605	(7.9)	902	(12.2)	38	(2.2)	67	(3)

*NST* no special type, *ILC* invasive lobular carcinoma, *MRI* magnetic resonance imaging, *n* number of patients, *¥* final operation, *BCS* breast conserving surgery, *NM* negative margin, *FPM* focal positive margin, *MFPM* more than focal positive margin

There were 2939 (9%) incomplete cases in the OS-cohort (most frequent missing case: histological grade (3.3%, 1060 observations)) and 231 (9.0%) in the DFS-cohort (most frequent missing case: histological grade (3.6%, 89 observations)) for which multiple imputation was used, assuming that the data were missing at random. The imputed data were compared with the original data, which showed an overall comparable distribution of the data (Online Appendix 1, Tables 1 and 2).

**Table 2:** Overview event and event type per (sub)group in DFS-cohort after 5 years

	Total study population		Invasive carcinoma NST		ILC	
	non-MRI (%)	MRI (%)	non-MRI (%)	MRI (%)	non-MRI (%)	MRI (%)
*Event*						
Yes	114 (6.5)	48 (6.9)	103 (6.3)	39 (7.3)	11 (7.9)	9 (5.5)
No	1653 (93.5)	649 (93.1)	1524 (93.7)	495 (92.7)	129 (92.1)	154 (94.5)
*Event type*						
LR	6 (0.4)	8 (1.2)	6 (0.4)	7 (1.3)	0	1 (0.6)
RR	10 (0.6)	2 (0.3)	10 (0.6)	2 (0.4)	0	0
DM	67 (3.8)	29 (4.2)	61 (3.7)	22 (4.1)	6 (4.3)	7 (4.3)
LR + RR	6 (0.4)	2 (0.3)	5 (0.3)	1 (0.2)	1 (0.7)	1 (0.6)
LR + DM	6 (0.4)	1 (0.1)	5 (0.3)	1 (0.2)	1 (0.7)	0
RR + DM	14 (0.8)	6 (0.9)	12 (0.7)	6 (1.1)	2 (1.4)	0
LR + RR + D M	5 (0.3)	0	4 (0.2)	0	1 (0.7)	0

*NST* no special type, *ILC* invasive lobular carcinoma, *MRI* magnetic resonance imaging, *LR* local recurrence, *RR* regional recurrence, *DM* distant metastasis

### Overall survival

**Fig. 1:**
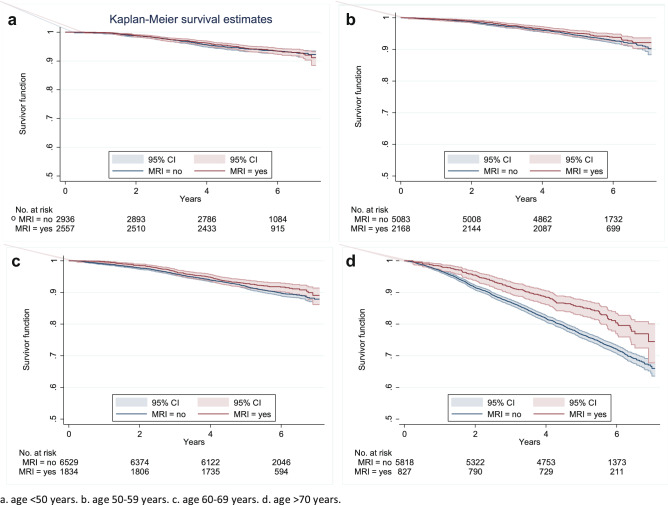
Kaplan–Meier curve of overall survivor function subgroup invasive carcinoma NST, stratified per age category (2011–2013, *n* = 27,752; *n*_a_ = 5493, *n*_b_ = 7251, *n*_c_ = 8363, *n*_d_ = 6645)

**Fig. 2:**
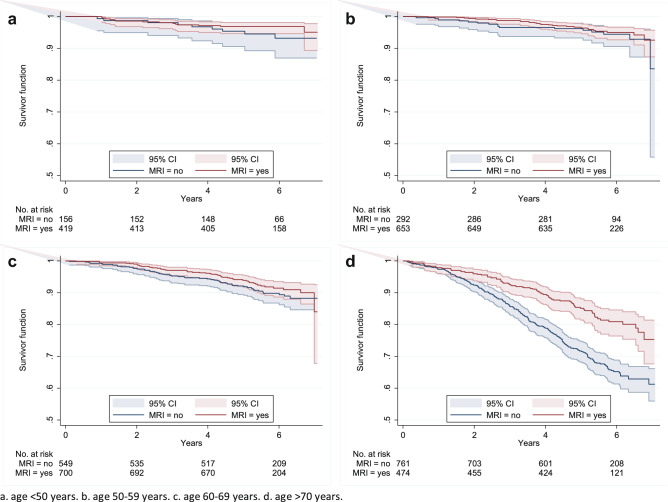
Kaplan–Meier curve of overall survivor function subgroup ILC, stratified per age category (2011–2013, *n* = 4004; *n*_a_ = 575, *n*_b_ = 945, *n*_c_ = 1249, *n*_d_ = 1235)

In the total OS-cohort (*n* = 31,756), 2938 (13.0%) of the 22,124 patients in the non-MRI group died, compared to 743 (8.0%) of the 9632 patients in the MRI group, after a mean follow-up of 5.3 years (range 0.1–7.1 years, see Table 3 in Online Appendix 2 for the events of deaths per subgroup, stratified by age categories). The Kaplan–Meier analysis and the log-rank test showed that patients in the MRI group had a significantly better OS than the patients in the non-MRI group (*p* < 0.0001); the 7-year survival rates were 0.89 (95%-CI 0.87–0.90) and 0.83 (95%-CI 0.82–0.83), respectively (Online Appendix 3). However, after age stratification in the total study population, this difference in OS proved to be only statistically significant for the age groups 60–69 (*p* = 0.036) and > 70 (*p* < 0.0001). In Figs. 1 and 2 the Kaplan–Meier curves are shown per histological subgroup and stratified by age category.

The multivariable analysis of the OS regarding breast MRI use demonstrated no statistical differences among different age categories for the effect of breast MRI, neither in the overall OS-cohort, nor in the histological subgroups (total study population: *p* = 0.06; invasive carcinoma NST: *p* = 0.07; ILC: *p* = 0.23) (Table 3).

**Table 3:** Results of multivariable Cox proportional hazard regression analysis, for both OS and DFS, respectively for total study population (a), invasive carcinoma NST (b) and ILC (c)

Variable	Overall survival			Disease-free survival		
	Total study population			Total study population		
	HR	95%-CI	*P*-value	HR	95%-CI	*P*-value
a						
*MRI*			–			–
Yes	0.91	(0.74–1.11)	0.35	1.16	(0.81–1.67)	0.42
*Age*			< 0.01*			0.29*
< 50	1	–		1	–	
50–59	3.52	(2.98–4.16)	< 0.01	1.35	(0.79–2.29)	0.27
60–69	1.25	(1.05–1.49)	0.01	1.55	(0.91–2.66)	0.11
> 70	1.76	(1.49–2.08)	< 0.01	1.83	(0.97–3.45)	0.06
*MRI#age*			0.06*			
< 50	1	–		–	–	
50–59	0.88	(0.67–1.17)	0.38	–	–	
60–69	0.88	(0.68–1.15)	0.36	–	–	
> 70	0.73	(0.57–0.93)	0.01	–	–	
*Tumour status*			< 0.01*			< 0.01*
1	1	–		1	–	
2	1.69	(1.56–1.83)	< 0.01	2.56	(1.74–3.78)	< 0.01
3–4	2.30	(1.99–2.66)	< 0.01	3.86	(2.00–7.46)	< 0.01
*Nodal status*			< 0.01*			< 0.01*
0	1	–		1	–	
1	1.66	(1.52–1.80)	< 0.01	1.70	(1.14–2.55)	0.01
2	3.46	(3.03–3.95)	< 0.01	4.75	(2.68–8.41)	< 0.01
3	6.01	(5.22–6.92)	< 0.01	10.59	(6.09–18.43)	< 0.01
*Hist. grade*			< 0.01*			< 0.01*
Low	1	–		1	–	
Medium	1.27	(1.14–1.41)	< 0.01	1.80	(0.96–3.36)	0.07
High	1.97	(1.75–2.21)	< 0.01	4.19	(2.20–7.97)	< 0.01
*Multifocal*						
Yes	£	–		£	–	
*Mol. subtype*			0.01*			0.53*
ER/PR + HER2−	1	–		1	–	
ER/PR + HER2 +	1.23	(1.06–1.42)	< 0.01	1.51	(0.78–2.90)	0.22
HER2 enr	1.41	(1.17–1.69)	< 0.01	1.68	(0.70–4.00)	0.25
Triple neg	1.74	(1.54–1.96)	< 0.01	1.26	(0.67–2.37)	0.48
*Tumour loc*			0.05*			
Lateral	1	–		£	–	
Medial	1.10	(1.01–1.21)	0.03	–	–	
Other	1.07	(1.00–1.16)	0.06	–	–	
*Final op*			–			
BCS	1.20	(1.07–1.34)	< 0.01	£	–	
* Surg. marg. ¥*			< 0.01*			
NM	1	–		£	–	
FPM	1.19	(1.02–1.38)	0.03	–	–	
MFPM	1.85	(1.43–2.40)	< 0.01	–	–	
* Adj. therapy*			< 0.01*			< 0.01*
Radio—yes	0.45	(0.40–0.50)	< 0.01	0.48	(0.34–0.69)	< 0.01
Chemo—yes	0.63	(0.57–0.70)	< 0.01	0.65	(0.38–1.11)	0.11
Horm.—yes	0.74	(0.67–0.81)	< 0.01	0.56	(0.33–0.97)	0.04
Target—yes	0.50	(0.41–0.62)	< 0.01	0.51	(0.23–1.10)	0.09

Variable	Overall survival			Disease-free survival		
	Invasive carcinoma NST			Invasive carcinoma NST		
	HR	95%-CI	*P*-value	HR	95%-CI	*P*-value
b						
*MRI*			–			–
Yes	0.96	(0.78–1.19)	0.74	1.23	(0.82–1.83)	0.32
*Age*			< 0.01*			< 0.01*
< 50	1	–		1	–	
50–59	1.28	(1.06–1.53)	< 0.01	1.30	(0.74–2.30)	0.36
60–69	1.79	(1.51–2.13)	< 0.01	1.78	(1.03–3.07)	0.04
> 70	3.49	(2.94–4.15)	< 0.01	2.67	(1.57–4.53)	< 0.01
*MRI#age*			0.07*			
< 50	1	–		–	–	
50–59	0.85	(0.63–1.14)	0.28	–	–	
60–69	0.83	(0.63–1.10)	0.19	–	–	
> 70	0.69	(0.53–0.91)	< 0.01	–	–	
* Tumour status*			< 0.01*			< 0.01*
1	1	–		1	–	
2	1.74	(1.60–1.89)	< 0.01	2.52	(1.70–3.76)	< 0.01
3–4	2.33	(1.97–2.76)	< 0.01	4.08	(1.90–8.78)	< 0.01
*Nodal status*			< 0.01*			< 0.01*
0	1	–		1	–	
1	1.66	(1.52–1.81)	< 0.01	1.57	(1.03–2.39)	0.03
2	3.55	(3.08–4.10)	< 0.01	4.09	(2.24–7.48)	< 0.01
3	5.98	(5.09–7.03)	< 0.01	8.86	(4.88–16.09)	< 0.01
* Hist. grade*			< 0.01*			< 0.01*
Low	1	–		1	–	
Medium	1.26	(1.13–1.41)	< 0.01	1.71	(0.86–3.42)	0.13
High	1.97	(1.74–2.23)	< 0.01	3.92	(1.97–7.80)	< 0.01
*Multifocal*						
Yes	£	–		£	–	
*Mol. subtype*			< 0.01*			0.40*
ER/PR + HER2−	1	–		1	–	
ER/PR + HER2 +	1.26	(1.08–1.47)	< 0.01	1.73	(0.88–3.37)	0.11
HER2 enr	1.43	(1.18–1.73)	< 0.01	1.68	(0.69–4.11)	0.26
Triple neg	1.71	(1.50–1.94)	< 0.01	1.18	(0.61–2.27)	0.62
*Tumour loc*			0.01*			
Lateral	1	–		£	–	
Medial	1.12	(1.02–1.23)	0.02	–	–	
Other	1.11	(1.02–1.20)	0.01	–	–	
*Final op*			–			
BCS	1.27	(1.12–1.43)	< 0.01	£	–	
* Surg. marg. ¥*			< 0.01*			
NM	1	–		£	–	
FPM	1.23	(1.05–1.45)	0.01	–	–	
MFPM	1.88	(1.38–2.56)	< 0.01	–	–	
*Adj. therapy*			< 0.01*			< 0.01*
Radio—yes	0.43	(0.38–0.48)	< 0.01	0.43	(0.30–0.63)	< 0.01
Chemo—yes	0.65	(0.58–0.73)	< 0.01	£	–	
Horm.—yes	0.74	(0.67–0.82)	< 0.01	0.57	(0.32–1.02)	0.06
Target—yes	0.45	(0.37–0.56)	< 0.01	0.44	(0.21–0.95)	0.04

Variable	Overall survival			Disease-free survival		
	ILC			ILC		
	HR	95%-CI	*P*-value	HR	95%-CI	*P*-value
c						
*MRI*			–			–
Yes	0.54	(0.23–1.24)	0.15	1.02	(0.36–2.94)	0.96
*Age*			< 0.01*			0.48*
< 50	1	–		1	–	
50–59	0.92	(0.40–2.10)	0.85	1.97	(0.36–10.82)	0.43
60–69	1.45	(0.71–2.98)	0.31	0.88	(0.14–5.40)	0.89
> 70	3.72	(1.83–7.56)	< 0.01	0.59	(0.09–3.73)	0.57
* MRI#age*			0.23*			
< 50	1	–		–	–	
50–59	1.67	(0.59–4.74)	0.33	–	–	
60–69	1.67	(0.66–4.19)	0.28	–	–	
> 70	1.10	(0.46–2.64)	0.84	–	–	
* Tumour status*			< 0.01*			0.31*
1	1	–		1	–	
2	1.39	(1.10–1.75)	< 0.01	2.83	(0.73–11.03)	0.13
3–4	2.02	(1.50–2.72)	< 0.01	2.82	(0.53–15.14)	0.23
*Nodal status*			< 0.01*			< 0.01*
0	1	–		1	–	
1	1.67	(1.33–2.11)	< 0.01	2.77	(0.72–10.66)	0.14
2	3.02	(2.09–4.36)	< 0.01	19.39	(3.16–118.88)	< 0.01
3	6.11	(4.59–8.13)	< 0.01	28.69	(5.75–143.08)	< 0.01
* Hist. grade*			< 0.01*			0.17*
Low	1	–		1	–	
Medium	1.28	(0.95–1.71)		2.57	(0.40–16.57)	0.32
High	1.92	(1.32–2.78)		6.86	(0.75–62.65)	0.09
* Multifocal*						
Yes	£	–		£	–	
*Mol. subtype*			0.04*			–
ER/PR + HER2−	1	–		1	–	
ER/PR + HER2 +	1.10	(0.66–1.84)	0.72	–	–	
HER2 enr	1.59	(0.67–3.78)	0.30	–	–	
Triple neg	1.85	(1.20–2.86)	< 0.01	1.08	(0.08–14.30)	0.95
*Tumour loc*						
Lateral	£	–		£	–	
Medial	–	–		–	–	
Other	–	–		–	–	
*Final op*						
BCS	£	–		£	–	
*Surg. marg. ¥*						
NM	£	–		1	–	
FPM	–	–		1.82	(0.34–9.75)	0.48
MFPM	–	–		11.91	(0.61–234.14)	0.10
*Adj. therapy*			< 0.01*			0.03*
Radio—yes	0.54	(0.45–0.66)	< 0.01	£	–	
Chemo—yes	0.56	(0.41–0.76)	< 0.01	0.20	(0.04–0.93)	0.04
Horm.—yes	0.73	(0.57–0.94)	0.01	0.31	(0.07–1.30)	0.11
Target—yes	£	–		£	–	

*NST* no special type, *ILC* invasive lobular carcinoma, *MRI* magnetic resonance imaging, *HR* hazard ratio, *¥* final operation, *MRI#age* interaction term MRI and age per category, only computed for OS-cohort, *Hist. grade* histological grade, *Mol. Subtype* molecular subtype, *HER2 enr.* HER2 enriched, *Triple neg.* triple negative, *Tumour loc.* tumour location, *Final op.* final operation, *BCS* breast conserving surgery, *Surg. Marg.* surgical margin, *NM* negative margin, *FPM* focal positive margin, *MFPM* more than focal positive margin, *Adj. therapy* adjuvant therapy, *Horm.* Hormonal

£ excluded due to Akaike’s information criterion selection method; *P-value overall likelihood ratio test

### Disease-free survival

Within the DFS-cohort (*n* = 2464), 114 (7.0%) of 1767 patients in the non-MRI group had an event compared to 48 (7.0%) of 697 in the MRI group, after a mean follow-up of 4.6 years. Table 2 provides an overview of the number of disease-related events after five years, according to MRI use. The Kaplan–Meier analysis and the log-rank test showed that undergoing a breast MRI was not significantly associated with DFS (*p* = 0.84). The 5-year DFS rates was 0.93 (95%-CI 0.91–0.95) for the MRI group vs 0.93 (95%-CI 0.92–0.94) in the non-MRI group (Online Appendix 3, Table 5). In both the invasive carcinoma NST and ILC subgroups, breast MRI use was also not significantly associated with DFS. In the invasive carcinoma NST group the 5-year survival rate was 0.93 (95%-CI 0.90–0.94) for the MRI group and 0.93 (95%-CI 0.92–0.95) for the non-MRI group (*p* = 0.54). In the ILC subgroup these figures were 0.94 (95%-CI 0.89–0.97) and 0.92 (95%-CI 0.86–0.95), respectively (*p* = 0.36).

The multivariable analysis showed no statistically significant difference in DFS regarding breast MRI use, neither in the total DFS-cohort, nor in the histological subgroups (total study population: *p* = 0.42; invasive carcinoma NST: *p* = 0.32; ILC: *p* = 0.96) (Table 3).

## Discussion

The purpose of this retrospective population-based study was to evaluate the association of breast MRI use on survival for invasive breast cancer patients treated with primary surgery in the Netherlands. Multivariable Cox regression showed that the effect of breast MRI on OS is not statistical different among different age categories (< 50 years, 50–59 years, 60–69 years and > 70 years, respectively), neither in the overall OS-cohort, nor in the histological subgroups. Yet, the use of breast MRI decreased dramatically from 49.0% in patients aged < 50 to 16.5% in patients aged > 70. Regarding the DFS-cohort, Multivariable Cox regression showed also no statistically significant difference in DFS between patients with or without breast MRI.

In contrast to our study, which demonstrated no significant effect of breast MRI use with regard to overall survival, a tendency towards positive correlation of breast MRI use on OS was observed in previous studies, but this association was not statistically significant [7, 11–14]. Ryu and colleagues indicated in a study of clinically T1-2 breast cancer patients that breast MRI use was not associated with a better OS (HR 1.18 95%-CI 0.27–5.08) [12]. Solin et al. indicated in a non-randomized retrospective analysis that there were no differences between the two groups for OS (univariable HR 0.84, 95%-CI 0.50–1.41, *p* = 0.51), however many patients underwent breast MRI after surgery within this study cohort [13]. In a non-randomized retrospective study towards early stage invasive carcinomas treated with breast conservation treatment (BCT), with a median follow-up of 13.8 years, Vapiwala et al. showed that breast MRI use had no significant impact on 15 year OS (MRI group 77% vs 71% non-MRI group, *p* = 0.24) [7]. Choi et al. found that the MRI group had a tendency towards better survival, however insignificant (univariable HR 0.79, 95%-CI 0.48–1.31, *p* = 0.362) [11]. Ha et al. studied the effect of breast MRI solely on ILC patients. Their results showed no statistical significance with the use of breast MRI or not regarding OS (HR 0.485, 95%-CI 0.149–1.585, *p* = 0.231) [14], which is in line with our results.

Although previously mentioned studies observed similar results when compared to our study, these studies were limited by heterogeneous data and several impairments. For instance multivariable analyses without including breast MRI [11], unequal follow-up time between both groups (MRI vs non-MRI) [12] or missing results of the multivariable models [13]. In addition, three of the previously mentioned studies focused solely on patients undergoing BCT [7, 12, 13] and two studies included both patients with breast conserving surgery as mastectomy [11, 14]. All studies were based on patients cohorts from single institutions, which may limit the generalizability of their findings [7, 11–14]. In addition, the study populations were significant smaller than our study population (range 287–2441) and patient characteristics were not completely balanced, the MRI group were younger and had slightly more favourable tumour characteristics, indicating that our study population seems a more balanced study population when compared to previously performed studies [7, 13, 14].

Surgical margin in patients treated with breast conservative surgery is an important prognostic factor as it is known to affect DFS in women who did not undergo breast MRI [9]. According to our results, the use of breast MRI resulted in statistically significant more frequent cancer-free surgical margins in the case of ILC (79% versus 73%, *p* = 0.002), but not in the subgroup of invasive carcinoma NST (*p* = 0.39). In contrast to our results, Lai et al. showed an overall increased risk of surgical margin involvement when breast MRI was omitted [18]. This could be explained, among other things, by the inclusion of patients diagnosed with DCIS only in the study of Lai et al. in contrast to our cohort including patients diagnosed with invasive breast cancer only. Consequently, the percentages of radical surgical margins differed among the studies (88.7% [18] versus 83.9% in our study).

This study has several strengths and limitations. One strength is the use of a nationwide population-based cancer registry, which increases the generalizability of the results. This also led to a large sample size, which made stratification on the subgroups invasive carcinoma NST and ILC, and the age categories possible. However, since the sample size was large, the confidence intervals were relatively small which might lead to statistically significant results, yet not clinically relevant. It is important to take this in consideration while interpreting the results. Another strength of this study is that it includes several other prospective factors which may influence and adjust the HR of breast MRI, such as the tumour location, type of operation and surgical margins.

A limitation of this study is the retrospective and observational design. This reflects daily practice which leads to a large difference in distribution of breast MRI use among the different age categories in our study. The use of breast MRI decreased with increasing age, especially in patients 60–70 and > 70. The use of MRI might be affected by factors which we did not have any information on in the NCR, such as the presence of comorbid conditions in patients. Another important limitation is that the reason to perform an MRI and whether the MRI findings changed the original surgical treatment plan or not were unknown, which leaves room for confounding by indication. Besides, the number of surgical excisions to achieve negative margins per patient in the current study was unknown. Next, for this study patients treated with neo-adjuvant systemic therapy were excluded. Yet, this subgroup of patients is considered to be an important subgroup of patients with regard to potential change in treatment decision based on breast MRI findings [19].

In addition, the patient characteristics and treatment characteristics within our observational study were not well balanced. After further examination of the treatment characteristics of the OS study population, it was somewhat noticeable that patients in the total study population and in the age categories > 60 treated with mastectomy, were slightly more treated with adjuvant radiotherapy in the MRI group, compared to the non-MRI group. Patients in the age category of > 70 and treated with breast conserving surgery, were slightly less treated with adjuvant radiotherapy in the non-MRI group, compared to the MRI group. In both the invasive carcinoma NST subgroup and the ILC subgroup the same results applied, however in the invasive carcinoma NST subgroup this was less noticeable. In addition, in the ILC subgroup patients in the age category of < 50 and treated with breast conserving surgery, were slightly less treated with adjuvant radiotherapy in the non-MRI group, compared to the MRI group. Although we corrected for the unbalanced patient and treatment characteristics in our multivariable analysis, we still cannot exclude residual confounding.

Another limitation is the maximum follow-up of 7 years, which resulted in a mean follow-up of 5.3 years. A longer follow-up would have given more insight into the possible long-term impact of breast MRI on the OS, especially with regard to hormone receptor positive patients, and would have increased the statistical power to detect clinically relevant differences. In addition, the cause of death was unknown in the current study, consequently breast cancer related mortality cannot be evaluated in the current cohort. In order to provide a better recommendation for breast MRI in general use, it is recommended that a next study should focus on a longer follow-up period, including a breast cancer related mortality analysis.

Finally, our DFS-cohort consisted only of a relatively small proportion of the patients out of the OS-cohort. Due to limited resources active follow-up performed by the registrars of the NCR could only be accomplished for the patients diagnosed in the first quartile of 2012.

Future studies should investigate the effect on prognosis by comparing patients in whom MRI findings led to a change in treatment plan with those without treatment change and/or patients without MRI. For instance, the currently recruiting European wide MIPA trial could identify this specific subgroup of patients allowing to investigate prognosis in these patients into detail [20].

In summary, use of breast MRI showed no statistically significant effect regarding DFS nor OS for different age categories in patients treated with primary surgery.

## Electronic supplementary material

Below is the link to the electronic supplementary material.

Supplementary file1 (DOCX 67 kb)
