# Supplementary material for: The effect of breast MRI on disease-free and overall survival in breast cancer patients: a retrospective population-based study
Source: Breast Cancer Res Treat. 2020 Sep 15;184(3):951–63. doi: 10.1007/s10549-020-05906-w (PMC7655574; doi:10.1007/s10549-020-05906-w)
Supplement: Supplementary file 1 — Supplementary file1 (DOCX 67 kb) [file 10549_2020_5906_MOESM1_ESM.docx]

# **Supplemental material**

#### *Comparison imputed data*

Table 1: Comparison proportions original data vs imputed data, for both OS-cohort and DFS-cohort

|  | | Overall survival | | | Disease free survival | | |
| --- | --- | --- | --- | --- | --- | --- | --- |
| Variable | | Original | Imputed | Difference | Original | Imputed | Difference |
| Tumour size | 1 | 68.3 | 68.3 | 0.6 | 66.8 | 71.9 | 5.1 |
|  | 2 | 28.6 | 28.6 | -0.5 | 30.8 | 26.5 | -4.2 |
|  | 3-4 | 3.1 | 3.1 | -0.1 | 2.4 | 1.5 | -0.9 |
| Nodal status | 0 | 67.4 | 78.7 | 11.3 | 66.8 | 85.4 | 18.6 |
|  | 1 | 25.2 | 17.9 | -7.4 | 25.8 | 12.5 | -13.2 |
|  | 2 | 4.7 | 2.3 | -2.4 | 4.8 | 0.7 | -4.0 |
|  | 3 | 2.7 | 1.2 | -1.5 | 2.7 | 1.4 | -1.3 |
| Histological grade | Low | 24.5 | 32.9 | 8.4 | 25.8 | 31.0 | 5.1 |
|  | Medium | 46.6 | 44.4 | -2.2 | 45.4 | 47.0 | 1.5 |
|  | High | 28.9 | 22.7 | -6.1 | 28.8 | 22.1 | -6.7 |
| Multifocal | Yes | 85.4 | 88.8 | 3.3 | 85.5 | 87.3 | 1.8 |
|  | No | 14.6 | 11.2 | -3.3 | 14.5 | 12.7 | -1.8 |
| Molecular subtype | ER/PR+HER2- | 77.6 | 82.7 | 5.0 | 77.0 | 80.2 | 3.2 |
|  | ER/PR+HER2+ | 8.3 | 5.3 | -3.0 | 9.2 | 7.0 | -2.1 |
|  | HER2 enriched | 3.8 | 3.8 | -0.1 | 3.6 | 3.0 | -0.5 |
|  | Triple negative | 10.3 | 8.3 | -2.0 | 10.3 | 9.8 | -0.5 |
| Tumour location | Lateral | 47.8 | 47.7 | -0.2 | 47.7 | 50.5 | 2.8 |
|  | Medial | 20.8 | 20.5 | -0.3 | 20.5 | 18.3 | -2.1 |
|  | Other | 31.4 | 31.8 | 0.4 | 31.9 | 31.2 | -0.7 |
| Surgical margin | NM | 93.4 | 93.1 | -0.3 | 92.9 | 89.3 | -3.6 |
|  | FPM | 5.8 | 6.1 | 0.2 | 6.5 | 8.4 | 1.8 |
|  | MFPM | 0.8 | 0.9 | 0.1 | 0.6 | 2.3 | 1.7 |

Abbreviations: NM = negative margin; FPM = Focal positive margin; MFPM = more than focal positive margin

Table 2: Comparison means original data vs imputed data, for both OS-cohort and DFS-cohort

|  | Overall survival | | Disease free survival | |
| --- | --- | --- | --- | --- |
| Variable | Original | imputation | Original | imputation |
| Tumour size | 1.35 | 1.35 | 1.36 | 1.36 |
| Nodal status | 1.43 | 1.42 | 1.43 | 1.43 |
| Histological grade | 2.04 | 2.04 | 2.03 | 2.03 |
| Multifocal | 0.15 | 0.15 | 0.14 | 0.14 |
| Molecular subtype | 1.47 | 1.47 | 1.47 | 1.47 |
| Tumour location | 1.84 | 1.84 | 1.84 | 1.84 |
| Surgical margin | 1.07 | 1.07 | 1.08 | 1.08 |

#### *Events per subgroup, stratified by age categories*

Table 3: Events of death stratified by age categories, OS-cohort (2011-2013)

|  | Total study population | | | | Invasive carcinoma NST | | | | ILC | | | |
| --- | --- | --- | --- | --- | --- | --- | --- | --- | --- | --- | --- | --- |
|  | Non-MRI | | MRI | | Non-MRI | | MRI | | Non-MRI | | MRI | |
| Age categories | n | (%)* | n | (%)* | n | (%)* | n | (%)* | n | (%)* | n | (%)* |
| <50 | 193 | (6.2) | 169 | (5.7) | 184 | (6.3) | 155 | (6.1) | 9 | (5.8) | 14 | (3.3) |
| 50-59 | 349 | (6.5) | 153 | (5.4) | 333 | (6.6) | 124 | (5.7) | 16 | (5.5) | 29 | (4.4) |
| 60-69 | 651 | (9.2) | 197 | (7.8) | 598 | (9.2) | 145 | (7.9) | 53 | (9.7) | 52 | (7.4) |
| >70 | 1,745 | (26.5) | 224 | (17.2) | 1,500 | (25.8) | 143 | (17.3) | 245 | (32.2) | 81 | (17.1) |

Abbreviations: NST = no special type; ILC = Invasive lobular carcinoma; MRI = Magnetic resonance imaging; n = Number of persons

* Percentage of deaths within whole age category

#### *Survival function and log-rank test*

Table 4: Overview total study population survival function OS per year, non-MRI vs MRI

|  | Non-MRI | | | | MRI | | | |
| --- | --- | --- | --- | --- | --- | --- | --- | --- |
| Year | Beginning total | Fail | Survivor function | 95%-CI | Beginning total | Fail | Survivor function | 95%-CI |
| 0 | 0 | 0 | 1.00 | . | 0 | 0 | 1.00 | . |
| 1 | 21813 | 299 | 0.99 | (0.98-0.99) | 9580 | 45 | 1.00 | (0.99-1.00) |
| 2 | 21277 | 516 | 0.96 | (0.96-0.97) | 9460 | 107 | 0.98 | (0.98-0.99) |
| 3 | 20691 | 569 | 0.94 | (0.93-0.94) | 9278 | 168 | 0.97 | (0.96-0.97) |
| 4 | 20070 | 599 | 0.91 | (0.91-0.91) | 9119 | 145 | 0.95 | (0.95-0.96) |
| 5 | 13661 | 521 | 0.88 | (0.88-0.89) | 6110 | 153 | 0.93 | (0.93-0.94) |
| 6 | 6836 | 314 | 0.86 | (0.85-0.86) | 3139 | 86 | 0.92 | (0.91-0.92) |
| 7 | 535 | 120 | 0.83 | (0.82-0.83) | 223 | 39 | 0.89 | (0.87-0.90) |

Abbreviations: OS = Overall survival; MRI = Magnetic resonance imaging; CI = Confidence interval

Table 5: Overview total study population survival function DFS per year, non-MRI vs MRI

|  | Non-MRI | | | | MRI | | | |
| --- | --- | --- | --- | --- | --- | --- | --- | --- |
| Year | Beginning total | Fail | Survivor function | 95%-CI | Beginning total | Fail | Survivor function | 95%-CI |
| 0 | 0 | 0 | 1.00 | . | 0 | 0 | 1.00 | . |
| 1 | 1720 | 17 | 0.99 | (0.98-0.99) | 693 | 1 | 1.00 | (0.99-1.00) |
| 2 | 1668 | 25 | 0.98 | (0.97-0.98) | 679 | 9 | 0.99 | (0.97-0.99) |
| 3 | 1596 | 36 | 0.95 | (0.94-0.96) | 651 | 16 | 0.96 | (0.94-0.97) |
| 4 | 1545 | 18 | 0.94 | (0.93-0.95) | 635 | 10 | 0.95 | (0.93-0.96) |
| 5 | 1453 | 18 | 0.93 | (0.92-0.94) | 602 | 12 | 0.93 | (0.91-0.95) |

Abbreviations: DFS = Disease free survival; MRI = Magnetic resonance imaging; CI = Confidence interval

#### *Univariable Cox proportional hazard regression analysis*

Table 6: Results of univariable cox proportional hazard regression analysis, for both DFS and OS

|  | Disease-free survival | | | | | | Overall survival | | | | | |
| --- | --- | --- | --- | --- | --- | --- | --- | --- | --- | --- | --- | --- |
|  | Total study population | | Invasive carcinoma NST | | ILC | | Total study population | | Invasive carcinoma NST | | ILC | |
| Variable | HR | 95%-CI | HR | 95%-CI | HR | 95%-CI | HR | 95%-CI | HR | 95%-CI | HR | 95%-CI |
| **MRI** |  |  |  |  |  |  |  |  |  |  |  |  |
| Yes | ^£^1.03 | (0.74-1.45) | ^£^1.12 | (0.78-1.62) | 0.67 | (0.28-1.61) | ^**^0.57 | (0.52-0.61) | ^**^0.58 | (0.53-0.64) | ^**^0.41 | (0.34-0.50) |
| **Age** |  | ^**^ |  | ^**^ |  |  |  | ^**^ |  | ^**^ |  | ^**^ |
| <50 | 1 | - | 1 | - | 1 | - | 1 | - | 1 | - | 1 | - |
| 50-59 | 0.83 | (0.20-1.39) | 0.78 | (0.45-1.36) | 1.28 | (0.31-5.36) | 1.04 | (0.91-1.19) | 1.03 | (0.90-1.19) | 1.22 | (0.74-2.01) |
| 60-69 | 0.98 | (0.60-1.60) | 1.00 | (0.59-1.69) | 0.82 | (0.18-3.67) | ^**^1.53 | (1.35-1.73) | ^**^1.49 | (1.31-1.70) | ^*^2.19 | (1.39-3.45) |
| >70 | ^£*^2.11 | (1.35-3.31) | ^£*^2.15 | (1.33-3.46) | 1.91 | (0.51-7.21) | ^**^4.83 | (4.31-5.40) | ^**^4.62 | (4.11-5.19) | ^**^7.69 | (5.04-11.74) |
| **Tumour size** |  | ^**^ |  | ^**^ |  |  |  | ^**^ |  | ^**^ |  | ^**^ |
| 1 | 1 | - | 1 | - | 1 | - | 1 | - | 1 | - | 1 | - |
| 2 | ^**^4.04 | (2.88-5.65) | ^**^4.44 | (3.11-6.34) | 2.09 | (0.76-5.75) | ^**^2.54 | (2.37-2.72) | ^**^2.62 | (2.44-2.82) | ^**^2.18 | (1.78-2.66) |
| 3-4 | ^**^8.71 | (4.84-15.68) | ^**^13.48 | (6.81-26.66) | ^*^3.87 | (1.09-13.73) | ^£**^4.69 | (4.15-5.30) | ^£**^5.38 | (4.65-6.23) | ^**^3.86 | (3.01-4.95) |
| **Nodal status** |  | ^**^ |  | ^**^ |  | ^**^ |  | ^**^ |  | ^**^ |  | ^**^ |
| 0 | 1 | - | 1 | - | 1 | - | 1 | - | 1 | - | 1 | - |
| 1 | ^*^1.84 | (1.27-2.67) | ^*^1.86 | (1.25-2.76) | 1.79 | (0.60-5.32) | ^**^1.54 | (1.43-1.66) | ^**^1.53 | (1.41-1.66) | ^**^1.59 | (1.28-1.97) |
| 2 | ^**^4.60 | (2.80-7.57) | ^**^4.31 | (2.52-7.37) | ^£*^7.37 | (1.91-28.52) | ^**^2.93 | (2.61-3.28) | ^**^3.06 | (2.71-3.45) | ^**^2.21 | (1.58-3.09) |
| 3 | ^**^12.49 | (7.89-19.76) | ^**^12.46 | (7.59-20.47) | ^£**^13.41 | (3.90-46.08) | ^£**^5.45 | (4.86-6.11) | ^£**^5.17 | (4.53-5.91) | ^**^6.39 | (5.04-8.12) |
| **Hist. grade** |  | ^**^ |  | ^**^ |  |  |  | ^**^ |  | ^**^ |  | ^**^ |
| Low | 1 | - | 1 | - | 1 | - | 1 | - | 1 | - | 1 | - |
| Medium | ^*^2.09 | (1.16-3.79) | ^*^2.08 | (1.08-3.98) | 1.38 | (0.31-6.11) | ^**^1.49 | (1.35-1.64) | ^**^1.43 | (1.29-1.60) | ^*^1.51 | (1.14-2.00) |
| High | ^**^6.77 | (3.86-11.86) | ^**^7.33 | (4.02-13.37) | 3.34 | (0.62-18.07) | ^**^2.70 | (2.45-2.98) | ^**^2.74 | (2.47-3.04) | ^**^2.56 | (1.80-3.64) |
| **Multifocal** |  |  |  |  |  |  |  |  |  |  |  |  |
| Yes | 1.21 | (0.80-1.83) | 1.36 | (0.87-2.11) | 0.59 | (0.17-2.03) | ^*^1.10 | (1.01-1.21) | ^*^1.11 | (1.01-1.23) | 1.03 | (0.83-1.27) |
| **Mol. subtype** |  | ^**^ |  | ^**^ |  |  |  | ^**^ |  | ^**^ |  | ^**^ |
| ER/PR+HER2- | 1 | - | 1 | - | 1 | - | 1 | - | 1 | - | 1 | - |
| ER/PR+HER2+ | 1.51 | (0.90-2.52) | 1.67 | (0.99-2.82) | - | - | 1.00 | (0.88-1.13) | 1.02 | (0.89-1.16) | 1.02 | (0.62-1.69) |
| HER2 enr. | ^**^3.04 | (1.70-5.44) | ^**^3.22 | (1.79-5.81) | - | - | ^£**^1.75 | (1.51-2.02) | ^£**^1.78 | (1.54-2.06) | ^*^2.69 | (1.20-6.02) |
| Triple neg. | ^**^3.01 | (2.04-4.43) | ^**^3.21 | (2.15-4.81) | 1.78 | (0.24-13.33) | ^£**^2.52 | (2.32-2.74) | ^£**^2.55 | (2.34-2.77) | ^**^4.23 | (2.97-6.02) |
| **Tumour loc.** |  |  |  |  |  |  |  | ^**^ | ^*^ |  |  |  |
| Lateral | 1 | - | 1 | - | 1 | - | 1 | - | 1 | - | 1 | - |
| Medial | 1.07 | (0.70-1.64) | 1.13 | (0.72-1.76) | 0.63 | (0.14-2.93) | 1.00 | (0.91-1.09) | 1.01 | (0.92-1.11) | 0.93 | (0.71-1.22) |
| Other | 1.40 | (0.99-1.97) | 1.44 | (1.00-2.09) | 1.11 | (0.44-2.80) | ^**^1.18 | (1.09-1.26) | ^**^1.18 | (1.09-1.28) | 1.14 | (0.94-1.38) |
| **Final op.** |  |  |  |  |  |  |  |  |  |  |  |  |
| BCS | ^**^0.32 | (0.23-0.44) | ^**^0.29 | (0.21-0.41) | 0.65 | (0.26-1.63) | ^**^0.41 | (0.38-0.44) | ^**^0.41 | (0.39-0.44) | ^**^0.33 | (0.27-0.41) |
| **Surg. marg.** |  |  |  |  |  |  |  | ^**^ | ^**^ |  | ^*^ |  |
| NM | 1 | - | 1 | - | 1 | - | 1 | - | 1 | - | 1 | - |
| FPM | 0.66 | (0.31-1.41) | 0.55 | (0.22-1.33) | 1.49 | (0.34-6.46) | 0.90 | (0.77-1.04) | 0.92 | (0.79-1.08) | 0.76 | (0.51-1.15) |
| MFPM | ^£*^3.50 | (1.12-10.91) | ^£^2.81 | (0.71-11.11) | 8.16 | (1.00-66.83) | ^£**^2.16 | (1.67-2.80) | ^£**^2.01 | (1.49-2.72) | ^**^2.63 | (1.60-4.33) |
| **Adj. therapy** |  |  |  |  |  |  |  |  |  |  |  |  |
| Radio - yes | ^*^0.58 | (0.42-0.79) | ^*^0.50 | (0.36-0.70) | 1.45 | (0.56-3.77) | ^**^0.48 | (0.45-0.51) | ^**^0.47 | (0.44-0.50) | ^**^0.59 | (0.49-0.70) |
| Chemo - yes | 1.20 | (0.88-1.64) | 1.29 | (0.93-1.80) | 0.68 | (0.26-1.78) | ^**^0.56 | (0.52-0.61) | ^**^0.59 | (0.55-0.64) | ^**^0.39 | (0.31-0.49) |
| Horm. - yes | 0.90 | (0.66-1.22) | 0.89 | (0.64-1.24) | 0.90 | (0.33-2.48) | ^£**^0.84 | (0.78-0.89) | ^£**^0.82 | (0.77-0.88) | 0.87 | (0.71-1.05) |
| Target - yes | 1.21 | (0.73-1.99) | 1.24 | (0.75-2.07) | - | - | ^**^0.50 | (0.42-0.58) | ^**^0.48 | (0.40-0.56) | ^£^1.00 | (0.57-1.73) |

Abbreviations: NST = no special type; ILC = Invasive lobular carcinoma; HR = Hazard ratio; CI = Confidence interval; MRI = Magnetic resonance imaging; Hist. grade = Histological grade; Mol. Subtype = Molecular subtype; HER2 enr. = HER2 enriched; Triple neg. = Triple negative; Tumour loc. = Tumour location; Final op. = Final operation; BCS = Breast conserving surgery; Surg. Marg. = Surgical Margin; NM = Negative margin; FPM = Focal positive margin; MFPM = More than focal positive margin; Adj. therapy = Adjuvant therapy; Horm. = Hormonal.

^*^ p<0.05

^**^p<0.000

^£^ HR is not in line with the Kaplan-Meier curve. Hence, it must be interpreted with caution.
